# Large Outbreak of *Cryptosporidium hominis* Infection Transmitted through the Public Water Supply, Sweden

**DOI:** 10.3201/eid2004.121415

**Published:** 2014-04

**Authors:** Micael Widerström, Caroline Schönning, Mikael Lilja, Marianne Lebbad, Thomas Ljung, Görel Allestam, Martin Ferm, Britta Björkholm, Anette Hansen, Jari Hiltula, Jonas Långmark, Margareta Löfdahl, Maria Omberg, Christina Reuterwall, Eva Samuelsson, Katarina Widgren, Anders Wallensten, Johan Lindh

**Affiliations:** Umeå University, Umeå, Sweden (M. Widerström, M. Lilja, M. Ferm C. Reuterwall, E. Samuelsson);; Jämtland County Council, Östersund, Sweden (M. Widerström, M. Omberg);; Public Health Agency of Sweden, Soina, Sweden (C. Schönning, M. Lebbad, G. Allestam, B. Björkholm, A. Hansen, J. Långmark, M. Löfdahl, K. Widgren, A. Wallensten, J. Lindh);; Mid Sweden University, Östersund, (T. Ljung,);; Östersund Municipality, Östersund (J. Hitula); and Karolinska Institutet, Stockholm (J. Lindh)

**Keywords:** cryptosporidiosis/epidemiology, cryptosporidiosis/prevention and control, cryptosporidiosis/transmission, diarrhea, disease outbreaks, drinking water, molecular typing, questionnaires, risk factors, water microbiology, water supply, waste management, parasites, waterborne infections, *Cryptosporidium hominis* infection

## Abstract

In November 2010, ≈27,000 (≈45%) inhabitants of Östersund, Sweden, were affected by a waterborne outbreak of cryptosporidiosis. The outbreak was characterized by a rapid onset and high attack rate, especially among young and middle-aged persons. Young age, number of infected family members, amount of water consumed daily, and gluten intolerance were identified as risk factors for acquiring cryptosporidiosis. Also, chronic intestinal disease and young age were significantly associated with prolonged diarrhea. Identification of *Cryptosporidium hominis* subtype IbA10G2 in human and environmental samples and consistently low numbers of oocysts in drinking water confirmed insufficient reduction of parasites by the municipal water treatment plant. The current outbreak shows that use of inadequate microbial barriers at water treatment plants can have serious consequences for public health. This risk can be minimized by optimizing control of raw water quality and employing multiple barriers that remove or inactivate all groups of pathogens.

Protozoan parasites of the genus *Cryptosporidium* can cause gastrointestinal illness in humans and animals (*1*). Twenty-six species and >60 genotypes have been identified (*2*). *C. parvum* and *C. hominis* are the most prevalent species that infect humans (*1*,*3*). Cryptosporidiosis is transmitted mainly by the fecal–oral route, usually through oocyst-contaminated water or food or by direct contact with an infected person or animal (*2*). Infectivity is dose dependent and certain subtypes are apparently more virulent, requiring only a few oocysts to establish infection (*1*,*4*). In healthy persons, gastrointestinal symptoms usually resolve spontaneously within 1–2 weeks, although asymptomatic carriage can occur (*2*). Nonetheless, in immunocompromised patients, severe life-threatening watery diarrhea can develop (*2*). Information is limited regarding the long-term effects of *Cryptosporidium* infection (*3*,*5*,*6*).

The global incidence of cryptosporidiosis is largely unknown, although the disease was recently identified as one of the major causes of moderate to severe diarrhea in children <5 years of age in low-income countries (*7*). In Sweden, cryptosporidiosis has been a notifiable disease since 2004, and ≈150 cases (≈1.7/100,000 population/year) were reported annually until 2009. However, cryptosporidiosis is probably underreported, mainly because sampling from patients with gastrointestinal symptoms and requests for diagnostic tests are insufficient (*3*,*8*).

Because of some inherent characteristics of the pathogen, *Cryptosporidium* infection has critical public health implications for drinking water and recreational waters. The oocysts are excreted in large numbers in feces, can survive for months in the environment (*5*), and are resistant to the concentrations of chlorine commonly used to treat drinking water (*9*). The first reported outbreak of waterborne human cryptosporidiosis occurred in the United States in 1984 (*10*), and since then, numerous outbreaks involving up to hundreds of persons have been identified in several parts of the world (*11*,*12*). However, only a few very large outbreaks have been documented (*13*–*15*); the most extensive occurred in 1993 in Milwaukee, Wisconsin, USA, in which ≈400,000 persons were infected with *Cryptosporidium* oocysts by drinking water from a water treatment plant (WTP) (*14*). *Cryptosporidium* spp. are the predominant protozoan parasites causing waterborne outbreaks worldwide (*11*). In 2012, an increase in *Cryptosporidium* infections, particularly by *C. hominis* IbA10G2, was reported in Europe (*16*).

In Sweden, only 1 drinking water outbreak involving *Cryptosporidium* has been recognized (Y. Andersson, pers. comm.), and a *C. parvum* outbreak associated with fecal contamination of a public swimming pool occurred in 2002 and affected ≈1,000 persons (*17*). A study of *Cryptosporidium* species and subtypes isolated from samples from 194 patients in Sweden during 2006–2008 identified 111 *C. parvum* infections and 65 *C. hominis* infections. Most patients with *C. hominis* infection had been infected abroad, and only 3 were considered to have sporadic domestic infections (*3*). A recent investigation of *Cryptosporidium* in raw water from 7 large WTPs in Sweden (not including the WTP of interest in the present study) identified 23 (11.5%) of 200 positive samples containing 1–30 oocysts/10 L, although neither species nor subtypes were analyzed (*18*).

The city of Östersund is located in central Sweden and has a population of ≈60,000. The major WTP in Östersund (WTP-Ö) draws surface water from nearby Lake Storsjön and supplies drinking water to ≈51,000 of the city’s inhabitants. At the time of the onset of the outbreak reported here, the purification process at WTP-Ö included pre-ozonation, flocculation, and sedimentation, followed by rapid sand filtering and chloramination. WTP-Ö is situated 4 km upstream from the major wastewater treatment plant (WWTP-Ö) to ensure that the drinking water intake will not be affected by the wastewater outlet (Figure 1).

**Figure 1:**
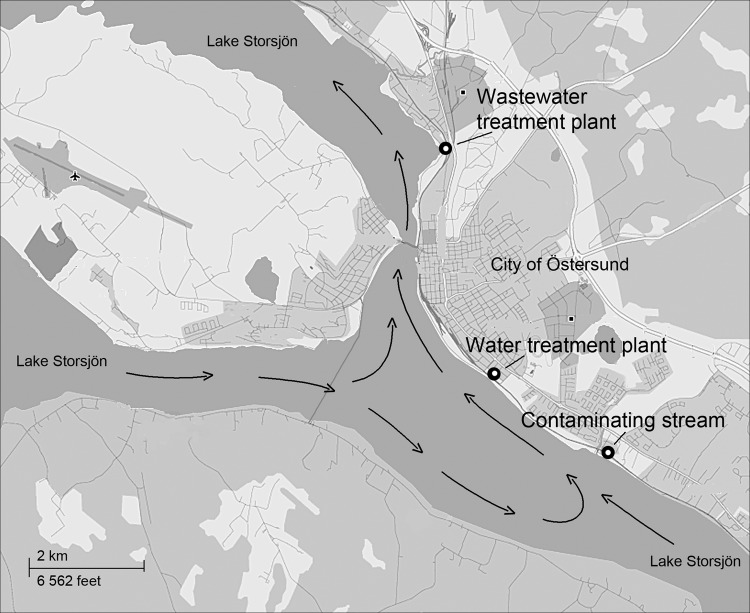
Map of Lake Storsjön, showing water currents (arrows) and locations of wastewater treatment plant, water treatment plant, and contaminating stream during *Cryptosporidium* infection outbreak, Östersund, Sweden, 2010–2011.

In late November 2010, the County Medical Office in Östersund received reports from several employers that 10%–20% of employees had gastroenteritis. The office advised that patients with acute gastroenteritis be tested for bacterial, viral, and protozoan pathogens. Among 20 patients from whom samples were obtained, 14 cases of cryptosporidiosis were detected on November 26. The local health advice line received numerous calls from persons with gastroenteritis, most of whom lived within the municipality (*19*). These facts indicated that the outbreak could be traced to the drinking water, and thus a boil-water advisory was issued for the municipality on November 26. This study describes the outbreak investigation and outlines the extent of the outbreak, clinical characteristics of persons infected, and risk factors for acquiring cryptosporidiosis.

## Methods

### Epidemiologic Investigation

#### Electronic Survey

To estimate the extent of the outbreak, the municipality published a questionnaire on its website during November 27–December 13, 2010. Persons in Östersund who had gastrointestinal symptoms were encouraged to provide information about day of onset, home address, and recent food intake. 

#### Written Questionnaire

Two months after the outbreak began, we conducted a retrospective cohort study, which included a random sample of 1,524 persons living in Östersund, to assess the extent of the outbreak, clinical characteristics of infected persons, and risk factors for acquiring cryptosporidiosis.

We estimated the proportion infected among the population of Östersund with a 3% margin of error (95% CI) by assuming a 50% attack rate and a 70% response rate when calculating the sample size. The patient questionnaire contained items on demographic characteristics, onset and occurrence of possible symptoms of cryptosporidiosis, water consumption, underlying diseases, and whether the WTP-Ö supplied water to the person’s workplace. Residential WTP supply was ascertained through population registers. Parents or guardians were asked to respond for children <15 years of age. A case-patient was defined as a person who lived in Östersund in mid-January 2011 and had had ≥3 episodes of diarrhea daily and/or watery diarrhea with onset after November 1, 2010, and before January 31, 2011. The study was approved by the Research Ethics Committee of the Faculty of Medicine, Umeå University, Umeå, Sweden.

### Microbiological Investigation

#### Human Samples

From November 1, 2010, through January 31, 2011, fecal samples from inhabitants of Östersund who had acute gastroenteritis were tested for various pathogens. *Cryptosporidium* oocysts were analyzed by standard concentration techniques and modified Ziehl-Neelsen staining (*20*); enteric bacterial pathogens by standard methods; noroviruses and sapoviruses by PCR; and *Entamoeba* spp. and *Giardia duodenalis* by conventional light microscopy.

#### Environmental Samples

During the outbreak, 163 samples of drinking water, raw water, and wastewater were collected to trace the source and monitor the presence of oocysts. Most water samples were collected at or near WTP-Ö and at WWTP-Ö. However, as the outbreak spread to nearby regions, sampling was also conducted at 14 other WTPs and 6 additional WWTPs. The municipality identified 4 different streams with high counts of *Escherichia coli* that may have contaminated the raw water, and samples from those streams were analyzed for *Cryptosporidium*. Also, as part of a then-ongoing national survey regarding presence of parasites in wastewater, 7 preoutbreak samples were collected at WWTP-Ö. The methods used are described in the Technical Appendix.

### Molecular Analysis/Typing

In a subset of fecal samples, *Cryptosporidium* species were determined by PCR–restriction fragment-length polymorphism analysis of the 18S rRNA gene (*21*). Species were further characterized by sequence analysis of the 60-kDa glycoprotein (*gp60*) gene (*22*). 

Oocysts in wastewater and stream water samples were isolated from the contaminating debris by immunomagnetic separation (IMS), and DNA was extracted (Technical Appendix). DNA was also extracted from oocysts that had been obtained from 1 raw water sample and 1 drinking water sample by use of Envirochek filters (Pall Life Science, Ann Arbor, MI, USA) followed by IMS. Microscope slides containing 1–13 oocysts from 4 raw water samples and 4 drinking water samples were sent to the Cryptosporidium Reference Unit, Swansea, United Kingdom (Technical Appendix), where molecular analyses were performed.

### Statistical Analysis

We conducted statistical analyses to test associations between risk factors and duration of diarrhea after controlling for age, sex, and residence in the area served by WTP-Ö. Student *t* test was used to analyze differences in attack rate and relapse rate. Relationships between risk factors and clinical cryptosporidiosis as the outcome variable were investigated by logistic regression. For dichotomous predictors, odds ratios were used to measure associations between clinical cryptosporidiosis and risk factors. Because of overdispersion in the data, negative binomial regression was applied to model the duration of infection in accordance with the case definition. Age and number of glasses of water consumed per day were evaluated as continuous variables. All statistical analyses were performed by using SPSS software version 19 (SPSS Inc., Chicago, IL, USA). A p value <0.05 was considered significant.

## Results

### Epidemiologic Investigation

#### Electronic Survey

**Figure 2:**
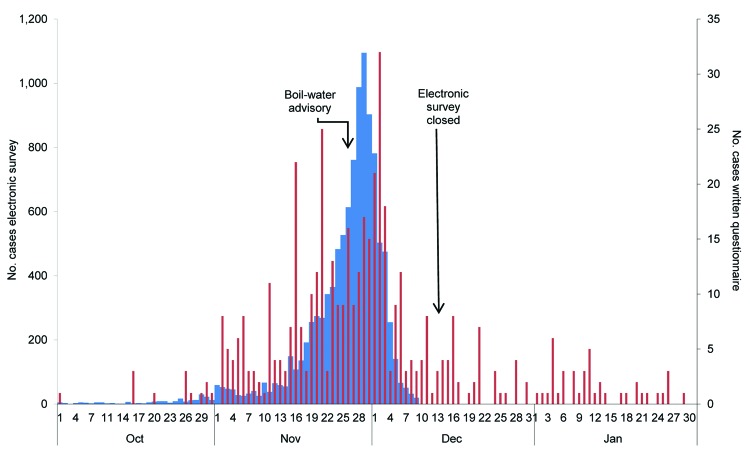
Epidemiologic curve of data from the electronic survey (10,653 participants; blue) and written questionnaire (434 participants; red) showing number of patients with suspected cases by date of onset of illness during *Cryptosporidium* infection outbreak, Östersund, Sweden, 2010–2011.

Gastrointestinal symptoms were reported by 10,653 persons over a period of 2.5 weeks, confirming the large outbreak in the city and contamination of the drinking water (Figure 2). The number of cases of gastrointestinal illness increased from mid-November and peaked on November 29, three days after the boil-water advisory was issued. Thereafter, the number of new cases reported per day rapidly declined.

#### Written Questionnaire

**Figure 3:**
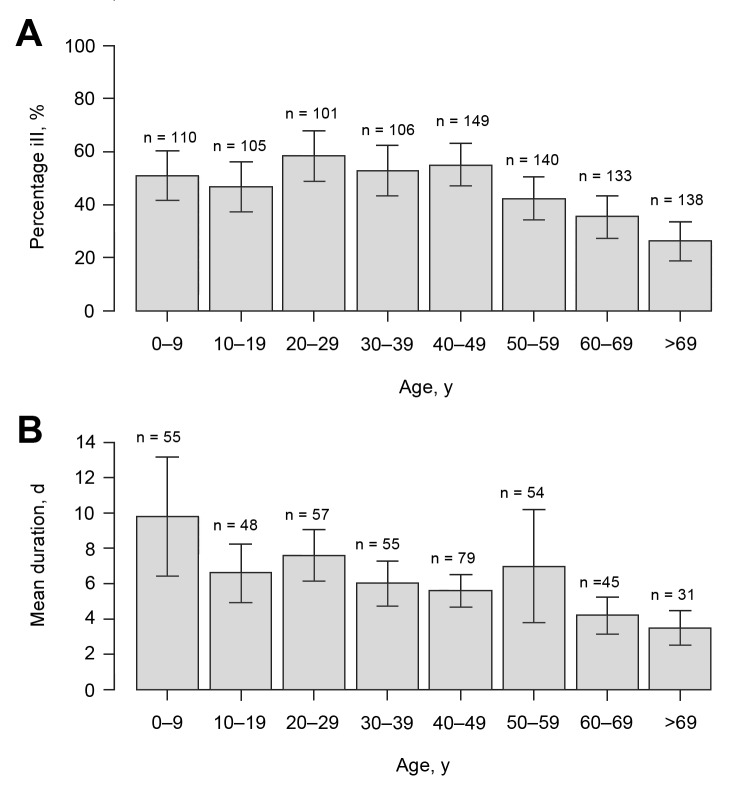
Percentage of ill persons (A) and mean duration of symptoms fulfilling the case definition (B), stratified by age group during *Cryptosporidium* infection outbreak, Östersund, Sweden, 2010–2011. Error bars represent ±1 SE.

Questionnaires were distributed by mail to 1,524 addressees; 10 persons had moved, and 6 were unable to respond. Of the remaining 1,508, a total of 1,044 (69.2%) responded: 481 men (46.1%) and 563 women (53.9%) (median age 44 years [range 0–98 years]) (Table 1). The response rate was highest for women 60–69 years of age (90.0%) and lowest for men 20–29 years (43.8%), and 45.2% (95% CI 42.1%–48.3%) of all the responders met the case definition criteria. When the rate of 45.2% was applied to the total population of Östersund (59,500), results indicated that ≈27,000 (95% CI 25,049–28,738) inhabitants contracted clinical cryptosporidiosis during the survey period. The attack rate decreased with age (p<0.0001; Table 1, Figure 3), was highest (58.0%) for persons 20–29 years of age and lowest (26.1%) for persons >69 years of age (Table 1), and was similar for men and women. The attack rate was 52.2% for respondents who lived and worked in areas served by the WTP-Ö but only 12.8% for inhabitants of Östersund who neither lived nor worked in areas served by that plant (p<0.0001; data not shown). The most common symptoms among case-patients were episodes of diarrhea >3 times daily (89.0%), watery diarrhea (84.3%), abdominal cramps (78.8%), fatigue (73.1%), nausea (63.9%), and headache (57.1%) (Table 2). Diarrhea lasted a median of 4 days (range 1–51 days). Duration of diarrhea decreased significantly with age (p<0.0001; Table 3, Figure 3), as did the incidence of fever, headache, nausea, vomiting, and fatigue (data not shown). Recurrence of diarrhea after >2 days of normal stools (defined as a relapse) was reported in 49.1% of the cases, and >1 relapse occurred significantly more often among women than men (p = 0.016; Table 4). Higher consumption of water and gluten intolerance were significant risks for *Cryptosporidium* infection (Table 3). Chronic intestinal disease (defined as inflammatory bowel disease [IBD], lactose intolerance, or gluten intolerance) and young age were significantly associated with more days with diarrhea (Table 3).

**Table 1:** Distribution of survey respondents and attack rate in *Cryptosporidium* infection outbreak, Östersund, Sweden, 2010–2011

Age group, y	No. respondents (%)				Attack rate %			
	All	Female	Male		All	Women	Men	p value
0–9	115 (67.3)	58 (67.4)	57 (67.1)		50.9	42.6	58.9	0.09
10–19	117 (66.5)	58 (61.1)	59 (72.8)		47.2	55.6	38.5	0.08
20–29	103 (48.8)	57 (53.8)	46 (43.8)		58.0	58.2	57.8	0.97
30–39	110 (55.8)	58 (60.4)	52 (51.5)		52.8	51.9	53.8	0.84
40–49	150 (66.7)	71 (70.3)	79 (63.7)		55.0	52.9	57.0	0.62
50–59	145 (79.2)	85 (84.2)	60 (73.2)		42.1	45.1	37.9	0.40
60–69	148 (89.2)	81 (90.0)	67 (88.2)		35.3	41.3	27.6	0.10
>69	156 (87.2)	95 (88.8)	61 (84.7)		26.1	24.4	28.8	0.57
Total	1,044 (69.2)	563 (72.0)	481 (66.3)		45.2	45.1	45.4	0.94

**Table 2:** Clinical characteristics of surveyed case-patients and non–case-patients in *Cryptosporidium* infection outbreak, Östersund, Sweden, 2010-2011

Symptom	No. positive answers/total no. respondents (%)*		
	All respondents, N = 972†	Case-patients, n = 434	Non-case-patients, n = 538
Diarrhea, >3 stools/d	382/967 (39.5)	382/429 (89.0)	0/538 (0)
Watery diarrhea	343/945 (36.3)	343/407 (84.3)	0/538 (0)
Abdominal cramps	382/952 (40.1)	328/416 (78.8)	54/536 (10.1)
Fatigue	342/921 (37.1)	302/413 (73.1)	40/508 (7.9)
Nausea	301/931 (32.3)	253/396 (63.9)	48/535 (9.0)
Headache	267/920 (29.0)	232/406 (57.1)	35/514 (6.8)
Fever >38.0°C	128/909 (14.1)	121/393 (30.8)	7/516 (1.4)
Muscle or joint aches	95/875 (10.9)	80/366 (21.9)	15/509 (2.9)
Vomiting	89/894 (10.0)	76/357 (21.3)	13/537 (2.4)
Eye pain	81/877 (9.2)	67/367 (18.3)	14/510 (2.7)
Bloody diarrhea	16/883 (1.8)	15/345 (4.3)	1/538 (0.2)

*Respondents who answered yes (case-patients) compared with those who answered no (non–case-patients) about whether they had experienced >3 episodes of diarrhea daily and/or watery diarrhea with onset after November 1, 2010. †Results on the basis of answers from 972 of 1,044 respondents.

**Table 3:** Risk factors for cryptosporidiosis and duration of infection in *Cryptosporidium* infection outbreak, Östersund, Sweden, 2010 –2011*

Risk factor	Infection†		Duration,‡ p value
	Adjusted OR (95% CI)	p value	
Age, continuous	0.99 (0.98–0.99)	<0.0001	<0.0001
Chronic intestinal disease§	1.86 (0.95–2.63)	0.08	<0.01
Chronic underlying disease#	1.15 (0.73–1.8)	0.55	0.59
Gluten intolerance	4.06 (1.24–13.29)	0.02	0.05
Lactose intolerance	1.40 (0.79–2.46)	0.25	<0.01
No. additional family members with cryptosporidiosis	1.99 (1.70–2.33)	<0.0001	NA
No. glasses of water consumed daily	1.07 (1.03–1.11)	<0.0001	0.07
No. persons in household	0.98 (0.87–1.07)	0.54	NA
Peptic ulcer or medication	1.26 (0.72–2.22)	0.42	0.43
Smoking	1.01 (0.58–1.75)	0.98	0.40

*OR, odds ratio, adjusted for age, sex, and residence in the water treatment plant area; NA, not applicable. †Participants with watery diarrhea and/or >3 episodes of diarrhea daily were defined as having cryptosporidiosis. ‡Duration (i.e., time fulfilling the case definition). §Defined as inflammatory bowel disease, lactose intolerance, or gluten intolerance. #Defined as cancer, rheumatic disease, cardiac failure, asthma, chronic obstructive pulmonary disease, or diabetes.

**Table 4:** Distribution of respondents and relapse of diarrhea among surveyed case-patients in the *Cryptosporidium* infection outbreak, Östersund, Sweden, 2010–11

Age group, y	All relapses, %	1 Relapse, %				>1 Relapse, %		
		Female	Male	p value		Women	Men	p value
0–9	68.5	50.0	43.8	0.66		22.7	21.9	0.94
10–19	48.9	20.7	50.0	0.04		20.7	10.0	0.30
20–29	40.4	22.6	19.2	0.76		22.6	15.4	0.50
30–39	47.3	25.9	32.1	0.63		29.6	7.1	0.03
40–49	51.3	27.8	36.4	0.42		25.0	13.6	0.21
50–59	47.4	22.2	23.8	0.89		25.0	23.8	0.92
60–69	47.8	22.6	20.0	0.85		29.0	20.0	0.52
>69	35.3	15.0	35.7	0.20		15.0	7.1	0.50
Total	49.1	25.4	33.5	0.07		24.1	15.0	0.016

### Microbiological Investigation

#### Human Samples

A total of 186 laboratory-confirmed cases of cryptosporidiosis related to the outbreak were reported to the national surveillance system: 149 in Jämtland County and 37 in other counties. Genotyping identified *C. hominis* subtype IbA10G2 in 37 samples. A representative sequence has been deposited into GenBank under accession no. KF574041. Analyses showed that the 149 *Cryptosporidum*-positive samples from Jämtland County were negative for other gastrointestinal pathogens.

#### Environmental Samples

*Cryptosporidium* oocysts were found in drinking water and raw water samples collected at the WTP-Ö on November 27 and in all samples of WTP-Ö drinking water, water from the distribution network, and raw water from Lake Storsjön over the next 2 months (Table 5). The highest number of oocysts in drinking water (1.4 presumptive oocysts/10 L) was detected on December 12, 2010 (Technical Appendix Figure 1). During the outbreak, the average oocyst density in drinking water was 0.32/10 L in WTP-Ö samples and 0.20/10 L in samples from the distribution network. Densities in raw water samples were generally higher: 0.2–3.1 oocysts/10 L. In WWTP-Ö wastewater, the pre-outbreak low density (<200 oocysts/10 L), had increased to 1,800/10 L on November 16, was highest at 270,000/10 L on November 29, and then gradually declined to preoutbreak levels from December 31 onward (Technical Appendix Figure 2).

**Table 5:** Presence of *Cryptosporidium* oocysts in environmental samples collected in Östersund, Sweden, November 27, 2010– March 22, 2011*

Sample type	No. samples	No. positive samples	Analyzed volume, L	Presumptive no. oocysts, min–max/10 L	Confirmed no. oocysts, min–max/10 L	Time span for positive samples
Raw water†	18	10	100	0.2–3.1	0.1–0.7	2010 Nov 27–2011 Feb 9
Drinking water, WTP-Ö†	7	7	800–1,500	0.047–1.4	0.02–1.3	2010 Nov 27–2011 Jan 20
Drinking water, distribution network	9	9	800–1,400	0.063–0.36	0.05–0.05	2010 Nov 29–2011 Jan 31
Wastewater, untreated†	21	13	0.05‡	200–270,000	§–160,000	2010 Nov 29–2011 Feb 17
Wastewater, treated	15	14	0.25–0.3‡	30–21,000	30–10,000	2010 Dec 1–2011 Jan 24
Recipient (Lake Storsjön)	14	8	9–10	2–21	1–18	2010 Nov 29–2011 Mar 22
Connected streams	8	5	2–10	1,300–5,000	950–3,500	2010 Nov 30–Dec 14
Other¶	10	2	10–17	1–3	1–3	2010 Nov 30–2011 Jan 17
Total	102	68		0.047–270,000	0.02–160,000	2010 Nov 27–2011 Mar 22

*Min, minimum; max, maximum; WTP-Ö, water treatment plant–Östersund.  †Details are available in Technical Appendix Figures 1 and 2. ‡These samples consisted of 30-mL aliquots from every 50–60 m^3^ of wastewater produced over 24 h. §Not possible to determine the lowest density by microscopy because of substantial background material in the concentrated water sample. ¶Samples from sources, such as swimming pools, water used to flush the distribution network, and sediment from fire hydrants.

Oocysts were detected in 4 of 22 raw water samples from other municipalities near Lake Storsjön but in only 1 drinking water sample from a WTP (Technical Appendix Table). All samples of untreated wastewater, most samples of treated wastewater (11/18), and samples from recipient water bodies (6/9) contained oocysts. Two of the 4 investigated streams connected to Lake Storsjön contained oocysts (Table 5). The stream closest to WTP-Ö (Figure 1) had densities of 1,300 and 5,000 oocysts/10 L on November 30 and December 2, respectively; this finding could be explained by wastewater leaking from an apartment building into the storm water system, which was repaired on December 3.

Isolated DNA from 1 concentrate of raw water, separated from other particulate matter by IMS, was successfully amplified at the 18S rRNA gene locus, and *C. hominis* was determined by restriction fragment length polymorphism and sequence analysis. Subtyping was not possible because amplification of the *gp60* gene failed. Also, despite repeated attempts, we were unable to amplify any DNA sequences from oocysts detected in raw water and drinking water by microscopy and removed from microscope slides.

*C. hominis* IbA10G2 was identified in 2 samples from the stream closest to WTP-Ö, in 5 from untreated wastewater at WWTP-Ö, and in 4 from other WWTPs in Jämtland County. No other *Cryptosporidium* species or subtypes were detected in any of the analyzed samples.

## Discussion

We describe a confirmed outbreak of *Cryptosporidium* infection affecting at least 27,000 inhabitants of Östersund, Sweden, which represents the largest known outbreak in Europe and the second largest worldwide after the Milwaukee outbreak. The etiologic agent was detected in drinking water, repeatedly over >2 months. Although *Cryptosporidium* spp. are occasionally found in untreated surface water, to our knowledge, this is the first time this pathogen has been detected in drinking water in Sweden.

Three factors facilitated detection of the outbreak. First, before the outbreak was recognized, alert staff at the county laboratory suspected oocysts in wet smears of unstained, concentrated fecal specimens and subsequently confirmed the presence of *Cryptosporidium* spp. by modified Ziehl-Neelsen staining, even though this analysis had not been specifically requested. Second, data from the local health advice line indicated that most persons with gastroenteritis resided within the city limits, which proved to be crucial for the decision to issue a boil-water advisory. Third, the electronic survey was a valuable tool for daily monitoring of the epidemic curve and evaluating the effect of the boil-water advisory. Previous research has demonstrated the benefits of event-based surveillance data and website questionnaires in early detection and monitoring of an outbreak (*23*,*24*).

The distribution of symptoms among case-patients with cryptosporidiosis in this study is comparable to observations from other studies (*6*,*17*,*25*), except regarding muscle or joint aches, which were reported less frequently in Östersund. Moreover, the median duration of diarrhea, the level of attack rates in different age groups, and recurrence rate of diarrhea correspond to findings in other outbreaks (*6*,*14*).

We identified young age, amount of water consumed, and number of infected family members as risk factors, which agrees with results from other studies (*26*,*27*). Also, gluten intolerance remained a risk factor after we controlled for age, sex, and residence in the WTP area, but this analysis was based on information from only 17 persons and hence should be interpreted with caution. The mechanism by which gluten intolerance might constitute a risk factor for cryptosporidiosis is unknown. Duration of diarrhea was significantly associated with young age and chronic intestinal disease. Exacerbation of IBD in cryptosporidiosis patients has been documented (*28*), and *Cryptosporidium*-induced loss of intestinal barrier function has been suggested to mimic changes seen in IBD (*29*). Additional studies are needed to clarify any long-term effects of *Cryptosporidium* infection and are being undertaken in relation to the current outbreak.

Molecular typing identified *C. hominis* IbA10G2 in both human and environmental samples. This early identification of non–livestock-associated *Cryptosporidium* isolates facilitated the outbreak investigation by indicating that the cause was contamination of surface water by human sewage rather than contamination from an animal source (*4*,*30*). *C. hominis* IbA10G2 is reported to be highly virulent; is excreted in high numbers in feces (*1*,*31*,*32*); and is the most commonly identified subtype in waterborne cryptosporidiosis outbreaks, including that in Milwaukee (*3*,*30*,*33*,*34*). These characteristics, along with occurrence of the outbreak in a population that may have been particularly susceptible because of limited previous exposure, contributed to the high attack rate (*35*,*36*).

Although the infectious dose for *Cryptosporidium* infection is low, the oocyst densities in the Östersund drinking water (maximum ≈1/10 L) cannot readily explain the high attack rate, even if the low recovery rate is taken into account. Densities may have been higher at the onset of the outbreak because of a surge of oocysts in the inlet before sampling, and secondary household transmission could have contributed to some of the cases. However, similar low numbers of oocysts have been detected in drinking water samples in other outbreaks (*26*,*37*). The level of recovery efficiency of the methods used in the outbreak required analysis of at least 100 L of water to identify the low level of *Cryptosporidium* contamination, which agrees with findings reported by other investigators (*26*).

Recovery studies were not performed during the acute phase of the Östersund outbreak, which underscores the uncertainty of extrapolating the numbers of oocysts detected in raw and drinking water to the actual density of oocysts (*38*). Moreover, no reliable assays to test viability and infectivity of oocysts are available (*1*). Other limitations of the present study include potential response bias in the electronic survey and the mailed questionnaire (*39*). Moreover, we could not assess the contribution of secondary transmission to the attack rate or ascertain the number of subclinical cases by serologic testing.

Several possible factors could explain *Cryptosporidium* contamination of the drinking water. In the routine bacteriologic analysis performed weekly at WTP-Ö, *E. coli* densities were ≈10 times greater than the average level on 3 occasions a few weeks before the outbreak (H. Dahlsten, pers. comm.), which implies abnormally high fecal contamination of the source water. Furthermore, *Cryptosporidium* oocysts were detected repeatedly in both raw and drinking water for months after the outbreak peaked, which illustrates the environmental persistence of oocysts and/or continuing contamination. Survival of the oocysts in Lake Storsjön was probably prolonged because the outbreak occurred in winter when the lake was covered with ice. The municipality of Östersund made considerable efforts to trace the sources of *Cryptosporidium* contamination, and tentatively identified 2 streams, 1 of which was located closer to (upstream of) the raw water intake (Figure 1) and had higher densities of oocysts. However, we could not establish whether the initial input of oocysts to Lake Storsjön and the raw water intake had actually come from these streams, or whether it resulted from the outbreak itself. Perhaps these 2 streams contributed to a transmission cycle in which infectious persons were shedding oocysts into leaking wastewater that reached the raw water intake. Because only *C. hominis* IbA10G2 was identified in environmental samples, we suggest that the outbreak was caused by a single common source of contamination, although this hypothesis could not be definitively demonstrated.

Failure of the WTP-Ö and onset of the outbreak has several plausible explations. To our knowledge, no posttreatment contamination or extensive failures in the treatment processes occurred, and routine tests of the drinking water showed no increased levels of fecal indicator bacteria. The WTP-Ö had 2 microbiological barriers (ozonation and chloramination) as recommended by the drinking water regulations in Sweden for surface waterworks, but these barriers were simply inadequate to remove or inactivate the *Cryptosporidium* oocysts in the raw water. The long-term solution to reduce infective parasites in Östersund was to install a UV water disinfection system, which was done after the outbreak in December 2010. In addition, pipes were repeatedly flushed, and and further sampling was conducted to verify that no potentially viable oocysts remained in the distribution network.

Previous research has suggested that analysis of *Cryptosporidium* in wastewater can aid in early detection of an outbreak (*40*). In Östersund, the number of *Cryptosporidium* oocysts in influent wastewater increased slightly 10 days before the boil-water advisory (1,800 oocysts/10 L), which indeed implies that monitoring the level of oocysts in influent wastewater might facilitate early detection of an ongoing outbreak, although the cost of such an approach would render it impractical.

Six months after the outbreak in Östersund, another waterborne outbreak of *C. hominis* IbA10G2 infection occurred in the city of Skellefteå, 450 km northeast of Östersund, possibly because persons from that city had visited Östersund during the outbreak there and had subsequently spread *Cryptosporidium* oocysts on their return to Skellefteå. In Sweden, recommendations to prevent outbreaks of parasites include identifying and limiting sources of contamination of raw water in combination with sampling (100-L volumes). The awareness of parasites as a probable cause of waterborne outbreaks has increased tremendously in this country since these outbreaks, and many WTPs have evaluated the efficiency of their current barriers, for example, by quantitative microbial risk assessment.

This study has documented the largest outbreak of waterborne cryptosporidiosis in Europe, affecting ≈27,000 persons. *C. hominis* subtype 1bA10G2 was identified in clinical samples and in wastewater. Low levels of oocysts were repeatedly detected in drinking water for >2 months. Our results emphasize the value of assessing microbial risks in raw water and using multiple barriers in WTPs to substantially reduce or inactivate all groups of microorganisms, including parasites such as *Cryptosporidium* spp.

Technical Appendix Methods used in molecular analysis of *Cryptosporidium* oocysts in wastewater and stream water samples (mmunomagnetic separation and extraction of DNA). Technical Appendix Figure 1. Number of *Cryptosporidium* oocysts/10 L of raw water and drinking water at the wastewater treatment plant in Östersund, Sweden, November 27, 2010–February 23, 2011. Technical Appendix Figure 2.. Number of *Cryptosporidium* oocysts/10 L of untreated wastewater at the wastewater treatment plant in Östersund, Sweden, September 21, 2010–February 22, 2011. Technical Appendix Table. Presence of *Cryptosporidium* oocysts in environmental samples collected in municipalities near Lake Storsjön, Sweden, December 6, 2010– March 9, 2011.
