# Supplementary material for: Large Outbreak of Cryptosporidium hominis Infection Transmitted through the Public Water Supply, Sweden
Source: Emerg Infect Dis. 2014 Apr;20(4):581–9. doi: 10.3201/eid2004.121415 (PMC3966397; doi:10.3201/eid2004.121415)
Supplement: Technical Appendix — Methods used in molecular analysis of Cryptosporidium oocysts in wastewater and stream water samples (mmunomagnetic separation and extraction of DNA). Technical Appendix Figure 1. Number of Cryptosporidium oocysts/10 L of raw water and drinking water at the wastewater treatment plant in Östersund, Sweden, November 27, 2010–February 23, 2011. Technical Appendix Figure 2.. Number of Cryptosporidium oocysts/10 L of untreated wastewater at the wastewater treatment plant in Östersund, Sweden, September 21, 2010–February 22, 2011. Technical Appendix Table. Presence of Cryptosporidium oocysts in environmental samples collected in municipalities near Lake Storsjön, Sweden, December 6, 2010– March 9, 2011. [file 12-1415-Techapp-s1.pdf]

Article DOI: http://dx.doi.org/10.3201/eid2004.121415

Large Outbreak of *Cryptosporidium hominis* Infection Transmitted through the Public Water Supply, Sweden

Technical Appendix

Primary Concentration of Oocysts by Filtration or Centrifugation

Raw water and drinking water samples (100–1,000 L water) were analyzed for *Cryptosporidium* (and *Giardia*) according to the ISO-15553:2006 (*1*) standard with filtration through Envirochek HV capsule filters (Pall Life Science, Ann Arbor, MI, USA). Ten-liter grab samples from recipient water bodies (lakes and rivers receiving treated wastewater) and streams were filtered by using a GE Polycarbonate Filter (pore size 2.0 µM and Ø 293 mm) mounted in a disc filter holder (Millipore, 293 mm, Millipore Corp., Bedford, MA, USA). Wastewater from the wastewater treatment plant in Östersund (WWTP-Ö) was collected as 24-hour samples (30 mL from every 50–60 m^3), and grab samples were collected at other WWTPs. Portions of 500 mL were sent to Swedish Institute for Communicable Disease Control (SMI) for analysis. All wastewater samples were concentrated by centrifugation at 2,000 × g for 10 min; this was done on 50–100 mL of untreated wastewater and 300 mL of treated wastewater.

Isolation of Oocysts by Immunomagnetic Separation and Staining with Fluorescent Antibodies

Concentrated samples of raw water, drinking water, wastewater, and stream water were further separated from other particulates by immunomagnetic separation (IMS) using a DynaBeads GC-Combo Kit (Invitrogen, Dynal AS, Oslo, Norway) according to manufacturer's instructions. The slide was again air dried at room temperature and stained with fluorescent antibodies (Aqua-Glo G/C direct, Waterborne, Inc., New Orleans, LA, USA) and thereafter with DAPI (4′,6-diamidino-2-phenylindole) (Sigma) according to standard procedures.

Each water sample was processed by IMS according to pellet size: for untreated wastewater samples, aliquots of different sizes were analyzed; for most of the raw water and drinking water collected, a whole sample was processed in 1 tube. Identification of oocysts was considered confirmed if 4 stained nuclei were observed but was considered presumptive if fewer

were observed. In untreated wastewater samples, the presence of debris hampered identification by DAPI staining.

DNA Extraction

Concentrates from 1 raw water sample (100 L) and 1 treated drinking water sample (1,000 L) that had been filtered through Envirochek filters were subjected to DNA extraction following IMS instead of drying onto microscope slides. Two concentrated water samples (10 L each) from the stream closest to the water treatment plant in Östersund (WTP-Ö) and 5 wastewater samples from the WWTP-Ö and 4 from other WWTPs located in Jämtland County (50–100 mL each) were processed by IMS. Thereafter, each of the 50-µL filtrates with 50 µL 0.1 M HCl added was transferred to a new 1.5-mL Eppendorf tube and neutralized with 5 µL of 1 M NaOH. To disrupt oocysts, the supernatant was subjected to repeated (5 × 15 min) freeze–thaw cycles (–70°C, 56°C) in 180 µL of ATL buffer as stipulated in the QIAamp DNA Mini Kit (QIAGEN, Hilden, Germany). DNA extraction was done as outlined by the manufacturers, with the following modifications: only 50 µL of AE buffer was used in the elution step, and the elution time was prolonged to 5 min.

Cryptosporidium species in raw water, wastewater, and drinking water were determined by PCR–restriction fragment-length polymorphism (RFLP) analysis of the 18 S rRNA gene (*2*). Subtypes were characterized by sequence analysis of the 60-kDa glycoprotein (*gp60*) gene (*3*). The 18S rRNA product was sequenced only if amplification of the *gp60* gene failed.

Analyses Performed at the *Cryptosporidium* Reference Unit, United Kingdom

Cryptosporidium oocysts on microscope slides were sent to Dr. Rachel Chalmers at the Cryptosporidium Reference Unit, Swansea, United Kingdom, for molecular analyses. These oocysts originated from the WTP-Ö and were obtained in 2010 from the following: 4 raw water samples collected on November 27 and 29 and on December 8 and 12; 4 drinking water samples collected on November 29 (2 samples) and December 2 and 8. The slides were analyzed according to the scraping/extraction method previously described by Chalmers et al. (*4*), with one modification: 8 freeze–thaw cycles were performed in liquid nitrogen instead of 3 cycles in dry ice/methanol. Four replicate SSU rRNA gene PCRs were performed, as well as 2 replicate PCRs targeting the COWP gene (*5*).

Laboratory Performance of Recoveries

Average recoveries achieved by laboratory analysis of *Cryptosporidium* at SMI were 62% (± SD 12%) for membrane filtration (raw and drinking water), 43% (± SD 12%) for Envirochek filtered raw and drinking water, 25% (± SD 5%) for untreated wastewater and 39% (± SD 13%) for treated wastewater. These recovery efficiencies are comparable to those reported in previous studies and external quality control programs, as well as internal controls spiked with Color Seed (TCS Biosciences Ltd., Buckingham, UK).

References

1. International Organization for Standardization. ISO standard: water quality—isolation and identification of Cryptosporidium oocysts and Giardia cysts from water. ISO 15553:2006.

2. Xiao L, Singh A, Limor J, Graczyk TK, Gradus S, Lal A. Molecular characterization of Cryptosporidium oocysts in samples of raw surface water and wastewater. Appl Environ Microbiol. 2001;67:1097–101. PubMed http://dx.doi.org/10.1128/AEM.67.3.1097-1101.2001

3. Alves M, Xiao L, Sulaiman I, Lal AA, Matos O, Antunes F. Subgenotype analysis of Cryptosporidium isolates from humans, cattle, and zoo ruminants in Portugal. J Clin Microbiol. 2003;41:2744–7. PubMed http://dx.doi.org/10.1128/JCM.41.6.2744-2747.2003

4. Chalmers RM, Robinson G, Elwin K, Hadfield SJ, Xiao L, Ryan U, et al. Cryptosporidium sp. rabbit genotype, a newly identified human pathogen. Emerg Infect Dis. 2009;15:829–30. PubMed http://dx.doi.org/10.3201/eid1505.081419

5. Spano F, Putignani L, McLauchlin J, Casemore DP, Crisanti A. PCR-RFLP analysis of the Cryptosporidium oocyst wall protein (COWP) gene discriminates between C. wrairi and C. parvum, and between C. parvum isolates of human and animal origin. FEMS Microbiol Lett. 1997;150:209–17. PubMed http://dx.doi.org/10.1016/S0378-1097(97)00115-8

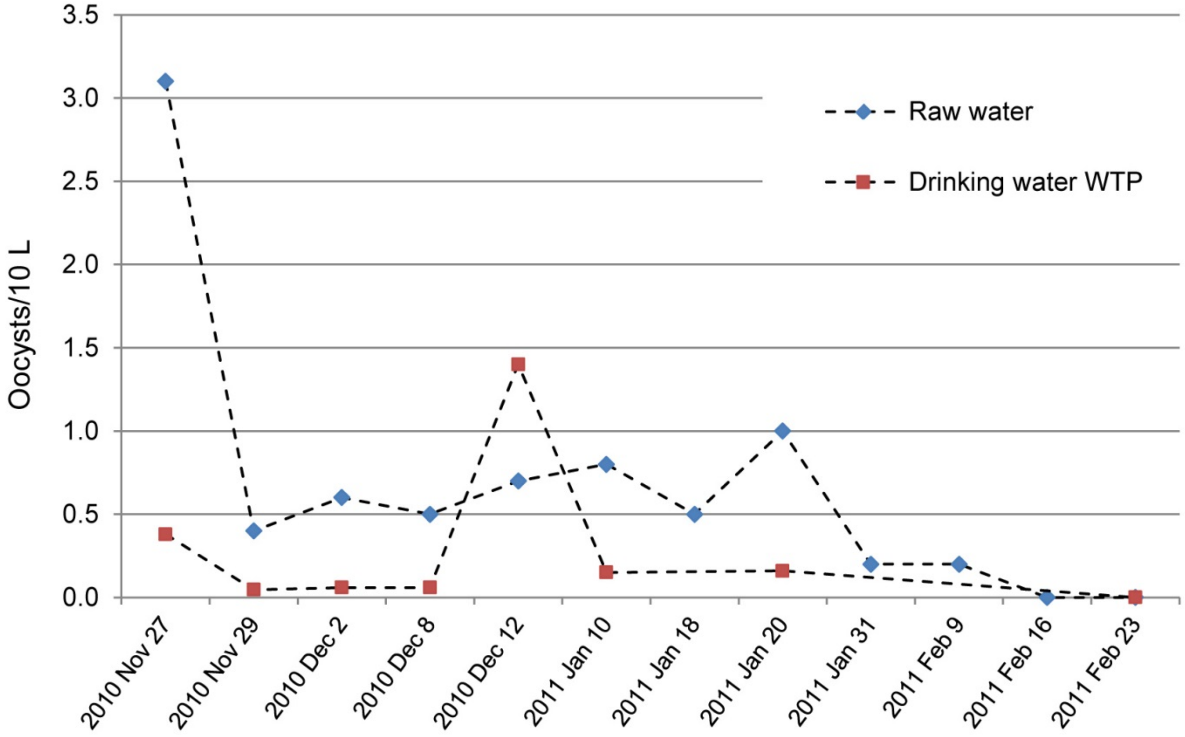

Technical Appendix Figure 1. Number of *Cryptosporidium* oocysts/10 L of raw water and drinking water at the wastewater treatment plant in Östersund, Sweden, November 27, 2010–February 23, 2011.

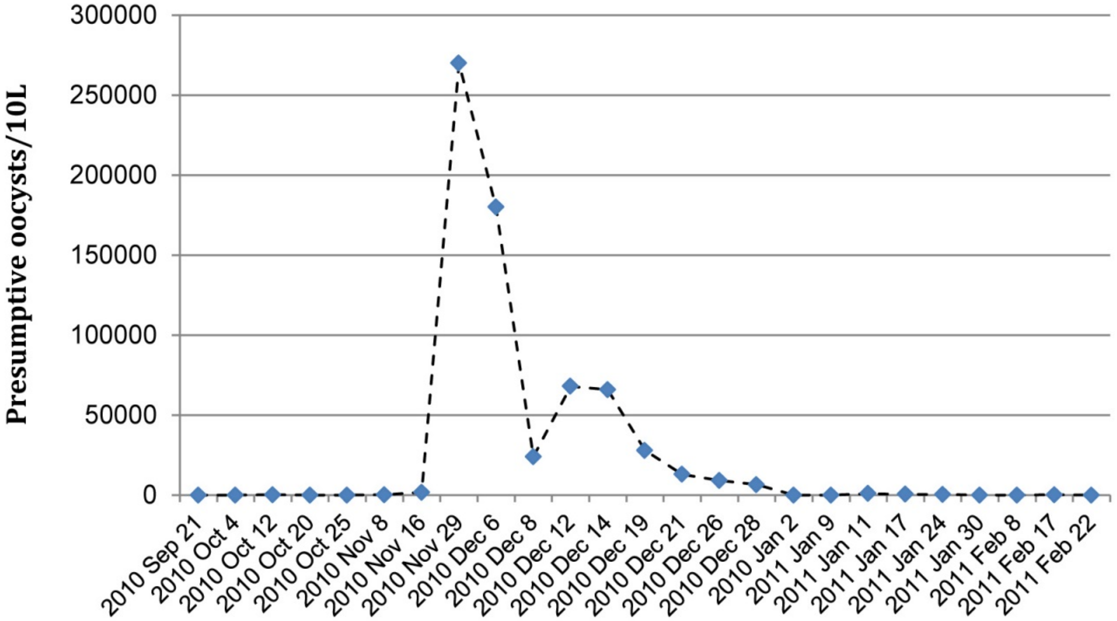

Technical Appendix Figure 2. Number of *Cryptosporidium* oocysts/10 L of untreated wastewater at the wastewater treatment plant in Östersund, Sweden, September 21, 2010–February 22, 2011.

Technical Appendix Table. Presence of *Cryptosporidium* oocysts in environmental samples collected in municipalities near Lake Storsjön, Sweden, December 6, 2010– March 9, 2011*

Type of sample	Total no. samples	No. positive samples	Analyzed volume (L)	Presumptive no. of oocysts min–max/10 L	Confirmed no. oocysts min–max/10 L	Time span for positive samples
Raw water	22	4	10–164	0.1–0.35	0.14– 0.29	2010 Dec 6–2011 Mar 9
Drinking water, WTPs	5	1	1,000–1,186	0.13	0.08	2010 Dec 9–2011 Feb 7
Drinking water, distribution network	1	0	1,000	NA	NA	2010 Dec 9
Wastewater, untreated	6	6	0.05‡	600–110,000	†	2010 Dec 13–2011 Jan 19
Wastewater, treated	18	11	0.25–0.3‡	30–6,100	30–2,800	2010 Dec 7–2011 Jan 30
Recipient water bodies	9	6	10	1–36	1–26	2010 Dec 14–Dec 27
Total	61	28		0.1–110,000	0.08–2,800	2010 Dec 6–2011 Mar 9

*Min, minimum; max, maximum; WTPs, water treatment plants; NA, not applicable.
†Not possible to determine density by microscopy because of substantial background material in the concentrated water sample.
‡Grab samples.
